# Supplementary material for: A Likelihood Approach for Real-Time Calibration of Stochastic Compartmental Epidemic Models
Source: PLoS Comput Biol. 2017 Jan 17;13(1):e1005257. doi: 10.1371/journal.pcbi.1005257 (PMC5240920; doi:10.1371/journal.pcbi.1005257)
Supplement: S1 File — (TAR.GZ) [file pcbi.1005257.s014.tar.gz › HSPH_Online-SI-Revision/IPoi09/n1-extreme/n1-extreme_BENCH_table4.pdf]

1	R0	{2.95019, 14.2854}	{2.86193, 14.6465}	{{2.86193, 14.6465}, {2.86193, 14.6465}}	{2.99167, 2.52776}	inf	101.	113.656	{63., 162.}	37.6887	0.144667	newly	40.	46.9344	{27., 71.}	42.2541	0.157433	Reff	0.31272	0.337755	{0.226092, 0.437875}	26.7333	ar	924.	929.213	{915., 944.}	1.20999
2	R0	{2.67565, 16.946}	{2.81635, 19.898}	{{2.81635, 19.898}, {2.81635, 19.898}}	{5.25854, 17.42}	inf	81.	133.738	{85., 198.}	67.1322	0.201262	newly	24.	43.2295	{23., 70.}	84.3579	0.237346	Reff	0.294322	0.431686	{0.259104, 0.566086}	52.8546	ar	926.	924.623	{907., 943.}	1.19322
3	R0	{2.40066, 16.6543}	{2.82135, 19.9006}	{{2.81635, 19.898}, {2.81635, 19.898}}	{17.5239, 19.4921}	inf	164.	181.623	{124., 243.}	23.5606	0.0956724	newly	51.	59.6885	{37., 86.}	30.2154	0.116089	Reff	0.40091	0.584814	{0.309799, 0.822374}	56.9632	ar	916.	924.984	{907., 942.}	1.44964
4	R0	{1.9294, 15.4361}	{2.78806, 19.8175}	{{2.7811, 19.6712}, {2.81635, 19.898}}	{44.5038, 28.3838}	inf	105.	156.954	{93., 221.}	54.4615	0.176515	newly	40.	49.9231	{26., 76.}	38.5769	0.140768	Reff	0.677219	0.505552	{0.284451, 0.756459}	45.3818	ar	796.	921.585	{902., 939.}	15.777
5	R0	{2.92859, 19.5393}	{1.30222, 1.35944}	{{1.30222, 1.35944}, {1.30222, 1.35944}}	{55.5342, 93.0425}	inf	114.	0.	{0., 0.}	100.	3.0569	newly	40.	0.	{0., 0.}	100.	2.60206	Reff	1.15316	1.03737	{0.5, 0.5}	200.	ar	927.	1.	{1., 1.}	99.8921
6	R0	{1.79622, 12.3148}	{2.6432, 19.8245}	{{2.50427, 19.3027}, {2.66225, 19.9254}}	{47.1536, 60.9808}	inf	56.	135.308	{75., 212.}	141.841	0.351454	newly	31.	42.9231	{22., 70.}	52.4566	0.17829	Reff	0.661009	0.478526	{0.300834, 0.713807}	40.0114	ar	734.	906.923	{890., 929.}	23.559
7	R0	{2.45855, 17.1776}	{2.88321, 17.0922}	{{2.80057, 16.2348}, {2.98125, 18.4042}}	{17.273, 5.30147}	inf	121.	111.571	{53., 171.}	33.5537	0.16513	newly	52.	40.2714	{19., 62.}	36.511	0.203561	Reff	0.486793	0.310157	{0.210726, 0.43926}	40.3063	ar	871.	931.671	{910., 948.}	6.96572
8	R0	{2.20009, 15.519}	{2.42205, 19.4475}	{{2.42205, 19.4475}, {2.42205, 19.4475}}	{10.0887, 25.3141}	inf	65.	78.4407	{32., 153.}	53.4289	0.20322	newly	25.	26.6949	{9., 53.}	49.3559	0.218529	Reff	0.448818	0.439458	{0.276114, 0.57887}	31.2359	ar	839.	879.458	{845., 908.}	4.94737
9	R0	{2.06493, 9.6414}	{2.85277, 14.9906}	{{2.84915, 14.6465}, {2.86193, 15.0981}}	{38.1533, 55.4814}	inf	54.	101.507	{43., 211.}	96.8544	0.250499	newly	29.	41.3014	{16., 91.}	67.265	0.213051	Reff	0.580245	0.325329	{0.205139, 0.490054}	49.1975	ar	777.	929.247	{914., 942.}	19.5942
10	R0	{2.60775, 19.6936}	{2.42205, 19.4475}	{{2.42205, 19.4475}, {2.42205, 19.4475}}	{7.12108, 1.24964}	inf	139.	93.7627	{39., 157.}	40.8609	0.257002	newly	45.	31.3898	{11., 58.}	43.5028	0.27516	Reff	0.47461	0.478375	{0.290646, 0.651531}	34.2337	ar	889.	879.458	{845., 908.}	2.02665
11	R0	{1.99168, 8.13555}	{2.85297, 15.1074}	{{2.84915, 15.0981}, {2.86193, 15.0981}}	{43.2446, 85.6964}	inf	45.	71.6875	{32., 137.}	74.3056	0.208996	newly	31.	28.5156	{12., 51.}	48.9415	0.235814	Reff	0.579579	0.269689	{0.188044, 0.381786}	53.6791	ar	772.	929.766	{913., 947.}	20.436
12	R0	{1.79304, 9.18941}	{2.7948, 15.8668}	{{2.78517, 15.7559}, {2.80057, 16.2348}}	{55.8695, 72.664}	inf	52.	154.508	{90., 226.}	197.132	0.448711	newly	36.	58.8983	{33., 91.}	68.7853	0.210202	Reff	0.627564	0.44293	{0.284087, 0.548678}	42.4495	ar	725.	925.22	{906., 943.}	27.6166
13	R0	{1.84016, 18.3431}	{2.26602, 19.3553}	{{2.26161, 19.3211}, {2.27287, 19.3849}}	{23.1423, 5.51801}	inf	88.	109.	{56., 167.}	39.8845	0.147851	newly	32.	37.1475	{17., 62.}	39.8566	0.155709	Reff	0.875916	0.559305	{0.366381, 0.823226}	44.7882	ar	762.	859.623	{836., 884.}	12.8114
14	R0	{1.94221, 9.85971}	{2.98732, 18.8837}	{{2.96797, 18.4042}, {2.99088, 18.9406}}	{53.8105, 91.5235}	inf	31.	94.52	{47., 180.}	205.075	0.431808	newly	20.	30.6533	{15., 60.}	66.0667	0.196803	Reff	0.42146	0.282469	{0.197398, 0.361896}	35.71	ar	805.	936.84	{924., 950.}	16.3776
15	R0	{1.78578, 12.0541}	{2.26184, 19.3836}	{{2.26161, 19.3849}, {2.26161, 19.3849}}	{26.6581, 60.8052}	inf	61.	108.161	{56., 167.}	81.9379	0.240401	newly	32.	35.9107	{17., 58.}	35.8817	0.147176	Reff	0.558949	0.553317	{0.343765, 0.823226}	31.4068	ar	732.	860.821	{840., 884.}	17.5986
16	R0	{1.94463, 15.6503}	{2.84207, 16.5048}	{{2.78517, 15.7559}, {2.96398, 17.7348}}	{46.1496, 5.71878}	inf	82.	75.8684	{38., 125.}	37.9974	0.184439	newly	29.	27.3158	{13., 48.}	39.6552	0.197447	Reff	0.663119	0.268954	{0.190997, 0.334443}	59.4411	ar	757.	930.066	{910., 946.}	22.8621
17	R0	{2.08901, 16.4314}	{2.64752, 19.8182}	{{2.64373, 19.7855}, {2.66225, 19.9254}}	{26.7357, 20.6117}	inf	116.	155.3	{86., 239.}	48.1404	0.16547	newly	42.	51.8714	{26., 85.}	46.3605	0.17271	Reff	0.676839	0.571734	{0.31196, 1.00101}	41.9022	ar	826.	907.571	{892., 926.}	9.87548
18	R0	{2.76419, 18.56}	{2.98031, 18.6427}	{{2.96797, 18.4042}, {2.99088, 18.9406}}	{7.81852, 1.51104}	inf	153.	116.913	{63., 218.}	38.2684	0.212537	newly	51.	38.7681	{18., 72.}	39.784	0.223955	Reff	0.351052	0.326159	{0.216662, 0.478541}	28.6965	ar	920.	934.841	{914., 949.}	1.96912
19	R0	{2.3821, 17.1399}	{2.84778, 16.5671}	{{2.78517, 15.7559}, {2.96797, 18.4042}}	{19.549, 5.41828}	inf	162.	107.	{49., 168.}	43.0795	0.264541	newly	73.	39.7183	{18., 68.}	51.7268	0.348782	Reff	0.443071	0.319924	{0.217043, 0.462338}	36.7216	ar	883.	928.197	{910., 946.}	5.16326
20	R0	{2.78314, 19.6127}	{2.50496, 19.1624}	{{2.50427, 19.1373}, {2.50427, 19.1373}}	{9.99508, 2.3599}	inf	71.	91.377	{43., 158.}	49.8961	0.170784	newly	24.	30.4918	{14., 56.}	51.5027	0.178399	Reff	0.222651	0.40704	{0.277974, 0.558452}	82.8151	ar	943.	892.23	{871., 909.}	5.38393
21	R0	{1.85124, 14.6794}	{2.43584, 19.3664}	{{2.42205, 19.1373}, {2.50427, 19.4475}}	{31.5788, 31.929}	inf	108.	155.692	{101., 214.}	49.3447	0.167285	newly	46.	54.0462	{32., 72.}	32.2074	0.125966	Reff	0.794182	0.689217	{0.3891, 1.3176}	37.6583	ar	730.	879.692	{859., 901.}	20.5058
22	R0	{2.1028, 14.9852}	{2.98037, 18.8611}	{{2.96797, 18.4249}, {2.99088, 18.9406}}	{41.7332, 25.8652}	inf	84.	75.0649	{30., 152.}	45.9184	0.22827	newly	32.	24.1299	{11., 46.}	45.8604	0.266407	Reff	0.471027	0.257223	{0.184126, 0.337969}	46.5526	ar	840.	936.753	{921., 949.}	11.5182
23	R0	{1.86601, 10.2157}	{2.97732, 18.6091}	{{2.96797, 18.1089}, {2.99088, 18.9406}}	{59.5552, 82.1617}	inf	60.	142.959	{80., 219.}	138.265	0.34555	newly	37.	49.0137	{27., 75.}	45.0574	0.151801	Reff	0.649371	0.358715	{0.223594, 0.484523}	48.7767	ar	747.	934.959	{917., 947.}	25.1618
24	R0	{2.34032, 10.0992}	{2.86193, 14.6465}	{{2.86193, 14.6465}, {2.86193, 14.6465}}	{22.288, 45.0263}	inf	58.	113.656	{63., 162.}	97.7671	0.259387	newly	35.	46.9344	{27., 71.}	50.1171	0.164898	Reff	0.355729	0.337755	{0.226092, 0.437875}	26.4626	ar	887.	929.213	{915., 944.}	4.77018
25	R0	{1.91548, 15.3401}	{2.67814, 19.7268}	{{2.64373, 19.6712}, {2.79269, 19.9254}}	{39.8154, 28.596}	inf	102.	141.731	{80., 227.}	48.6099	0.162378	newly	45.	45.6716	{25., 75.}	35.1907	0.156491	Reff	0.739375	0.475568	{0.300475, 0.772052}	46.5935	ar	765.	911.418	{894., 932.}	19.1396
26	R0	{2.75283, 10.6721}	{2.97443, 18.3746}	{{2.96797, 18.1089}, {2.99088, 18.9406}}	{8.0498, 72.1744}	inf	40.	164.646	{97., 241.}	311.615	0.591762	newly	22.	56.2769	{31., 93.}	155.944	0.377498	Reff	0.286294	0.450342	{0.253774, 0.694505}	63.7225	ar	926.	937.554	{921., 950.}	1.62652
27	R0	{2.08367, 19.7849}	{2.49856, 19.168}	{{2.42205, 19.1373}, {2.50427, 19.3027}}	{19.9114, 3.11796}	inf	132.	92.0645	{43., 158.}	42.0577	0.248462	newly	42.	30.2742	{14., 47.}	43.9708	0.256266	Reff	0.614683	0.408212	{0.282983, 0.553444}	39.039	ar	833.	891.435	{871., 909.}	7.01506
28	R0	{2.64334, 17.1765}	{2.97795, 18.6278}	{{2.96797, 18.4042}, {2.99088, 18.9406}}	{12.6586, 8.44943}	inf	110.	150.441	{76., 271.}	50.8021	0.169051	newly	34.	51.6176	{26., 93.}	62.1972	0.186224	Reff	0.311914	0.402323	{0.226575, 0.650382}	45.0475	ar	934.	935.382	{914., 950.}	1.14309
29	R0	{2.87008, 13.3872}	{2.64669, 19.8079}	{{2.64373, 19.7855}, {2.66225, 19.9254}}	{7.7833, 47.9614}	inf	80.	136.7	{73., 231.}	76.625	0.215583	newly	36.	44.6429	{20., 85.}	54.3254	0.208517	Reff	0.226736	0.491603	{0.29081, 0.756107}	116.817	ar	941.	908.929	{894., 928.}	3.40823
30	R0	{2.06105, 19.6275}	{2.42024, 19.4471}	{{2.42205, 19.4475}, {2.42205, 19.4475}}	{17.4274, 0.91918}	inf	42.	53.069	{18., 103.}	66.6667	0.241784	newly	18.	16.9828	{5., 33.}	60.6322	0.306021	Reff	0.595643	0.38368	{0.256737, 0.481988}	42.3052	ar	747.	878.741	{845., 905.}	17.6361
31	R0	{2.30764, 13.435}	{2.81777, 16.2728}	{{2.78517, 15.7559}, {2.96797, 18.1089}}	{22.1062, 21.1221}	inf	82.	74.6	{33., 143.}	41.5197	0.204866	newly	42.	26.8923	{11., 50.}	46.3736	0.300072	Reff	0.376145	0.285292	{0.215644, 0.399488}	29.2014	ar	877.	924.046	{899., 939.}	5.39251
32	R0	{2.30565, 11.9237}	{2.93438, 17.9458}	{{2.80057, 16.2348}, {2.98558, 18.4749}}	{27.2691, 50.5052}	inf	65.	159.881	{93., 237.}	145.97	0.366006	newly	34.	55.8358	{33., 83.}	66.9447	0.202064	Reff	0.378127	0.393829	{0.246342, 0.590626}	34.5612	ar	853.	936.537	{922., 949.}	9.79335
33	R0	{2.24031, 18.582}	{2.42205, 19.4475}	{{2.42205, 19.4475}, {2.42205, 19.4475}}	{8.11227, 4.65773}	inf	156.	125.559	{61., 198.}	34.4415	0.192252	newly	47.	42.0169	{18., 68.}	38.5142	0.196172	Reff	0.578	0.589749	{0.334243, 0.905847}	39.5319	ar	864.	879.458	{845., 908.}	2.5973
34	R0	{2.33306, 15.9759}	{2.9852, 18.8776}	{{2.96978, 18.4249}, {2.99088, 18.9406}}	{27.9523, 18.1631}	inf	110.	153.325	{80., 269.}	50.3424	0.164206	newly	38.	51.7922	{27., 88.}	49.9658	0.162808	Reff	0.468945	0.388679	{0.227307, 0.595185}	35.7359	ar	854.	936.506	{923., 949.}	9.66118
35	R0	{2.48357, 18.3573}	{2.95762, 18.2305}	{{2.83341, 16.5525}, {2.99088, 18.9406}}	{19.0875, 2.11454}	inf	120.	128.347	{78., 192.}	30.8444	0.125548	newly	42.	42.0667	{24., 58.}	30.7619	0.13514	Reff	0.588606	0.315451	{0.213694, 0.436558}	47.2212	ar	835.	938.04	{927., 950.}	12.3401
36	R0	{2.59548, 19.9046}	{2.81635, 19.898}	{{2.81635, 19.898}, {2.81635, 19.898}}	{8.50979, 0.0331582}	inf	123.	133.738	{85., 198.}	30.3745	0.121902	newly	46.	43.2295	{23., 70.}	33.0364	0.157099	Reff	0.490546	0.431686	{0.259104, 0.566086}	38.5223	ar	863.	924.623	{907., 943.}	7.14055
37	R0	{2.29641, 16.9868}	{2.26161, 19.3849}	{{2.26161, 19.3849}, {2.26161, 19.3849}}	{1.51541, 14.1174}	inf	111.	93.5893	{42., 169.}	35.0869	0.184469	newly	31.	31.75	{12., 53.}	39.977	0.184368	Reff	0.420243	0.505551	{0.332457, 0.721454}	31.6386	ar	877.	860.304	{840., 884.}	2.21738
38	R0	{2.65743, 15.4569}	{2.81635, 19.898}	{{2.81635, 19.898}, {2.81635, 19.898}}	{5.98021, 28.7322}	inf	85.	111.082	{65., 186.}	43.1051	0.143356	newly	40.	35.5738	{21., 66.}	36.5574	0.177166	Reff	0.385327	0.373743	{0.236573, 0.456249}	32.8588	ar	897.	924.623	{907., 943.}	3.13431
39	R0	{2.67953, 14.5132}	{2.98807, 18.9185}	{{2.99088, 18.9406}, {2.99088, 18.9406}}	{11.5148, 30.3535}	inf	119.	123.	{70., 212.}	37.1103	0.15622	newly	46.	41.4516	{19., 74.}	40.8836	0.195979	Reff	0.404609	0.341745	{0.209362, 0.541349}	41.8427	ar	894.	938.887	{927., 953.}	5.02093
40	R0	{1.94594, 11.928}	{2.97615, 18.7398}	{{2.96797, 18.4042}, {2.99088, 18.9406}}	{52.9413, 57.1079}	inf	60.	88.7391	{40., 166.}	62.1014	0.189958	newly	31.	28.942	{13., 55.}	40.5797	0.189592	Reff	0.558485	0.275919	{0.185435, 0.361896}	51.965	ar	796.	936.232	{921., 948.}	17.6171
41	R0	{1.66297, 10.0047}	{2.26161, 19.3849}	{{2.26161, 19.3849}, {2.26161, 19.3849}}	{35.9982, 93.7579}	inf	53.	108.268	{58., 167.}	106.368	0.289295	newly	33.	35.8929	{17., 58.}	34.5238	0.146214	Reff	0.640243	0.553529	{0.352811, 0.823226}	34.1443	ar	716.	860.304	{840., 884.}	20.1541
42	R0	{2.91568, 16.4453}	{2.86193, 14.6465}	{{2.86193, 14.6465}, {2.86193, 14.6465}}	{1.84348, 10.9381}	inf	138.	83.6557	{47., 124.}	48.5745	0.301082	newly	55.	33.1803	{16., 54.}	50.2235	0.316225	Reff	0.297399	0.28929	{0.211783, 0.357741}	21.0591	ar	919.	929.213	{915., 944.}	1.4895
43	R0	{2.55898, 15.8889}	{2.98523, 18.7856}	{{2.96797, 18.4042}, {2.99088, 18.9406}}	{16.6569, 18.2309}	inf	147.	185.618	{105., 268.}	37.5134	0.135317	newly	62.	63.7632	{34., 101.}	31.6214	0.138967	Reff	0.455498	0.480914	{0.260207, 0.828474}	38.5941	ar	904.	936.026	{921., 950.}	3.60969
44	R0	{2.28649, 15.9017}	{2.83659, 19.9106}	{{2.81635, 19.898}, {2.93978, 19.9817}}	{24.0588, 25.2107}	inf	92.	157.565	{112., 221.}	72.0722	0.219676	newly	43.	49.3065	{35., 70.}	28.8447	0.110661	Reff	0.400136	0.482696	{0.284451, 0.673108}	39.0264	ar	880.	925.71	{909., 942.}	5.19428
45	R0	{2.65121, 18.6339}	{2.85554, 14.8563}	{{2.84915, 14.6465}, {2.86193, 15.0981}}	{7.70702, 20.2727}	inf	160.	127.448	{63., 249.}	41.3899	0.224617	newly	45.	52.1343	{25., 106.}	49.0879	0.18856	Reff	0.371169	0.382627	{0.227932, 0.637804}	36.7983	ar	928.	930.119	{916., 943.}	0.977869
46	R0	{2.52291, 18.0251}	{2.81635, 19.898}	{{2.81635, 19.898}, {2.81635, 19.898}}	{11.631, 10.3905}	inf	131.	111.082	{65., 186.}	34.4513	0.171858	newly	46.	35.5738	{21., 66.}	39.1304	0.211197	Reff	0.431418	0.373743	{0.236573, 0.456249}	35.7643	ar	894.	924.623	{907., 943.}	3.45839
47	R0	{2.22618, 17.1225}	{2.83626, 19.8861}	{{2.81635, 19.898}, {2.86656, 19.8999}}	{27.4047, 16.14}	inf	148.	174.577	{113., 243.}	29.587	0.112623	newly	59.	56.3099	{33., 82.}	26.4264	0.123374	Reff	0.634461	0.53482	{0.295717, 0.904048}	44.4599	ar	850.	927.296	{914., 941.}	9.09362
48	R0	{2.04405, 16.073}	{2.88167, 18.9994}	{{2.81635, 18.33}, {2.97157, 19.9817}}	{40.9785, 18.2072}	inf	103.	124.92	{74., 194.}	36.7896	0.132884	newly	40.	40.7333	{24., 60.}	31.1	0.133006	Reff	0.711329	0.343812	{0.234546, 0.464688}	52.934	ar	779.	929.707	{911., 949.}	19.3462
49	R0	{2.84396, 18.097}	{2.86824, 14.6038}	{{2.86193, 14.6465}, {2.86193, 14.6465}}	{0.853781, 19.3025}	inf	132.	81.1148	{42., 124.}	49.0561	0.304055	newly	43.	31.8852	{15., 51.}	45.9016	0.256966	Reff	0.264488	0.280269	{0.194611, 0.354879}	20.8578	ar	952.	930.689	{917., 944.}	2.24893
50	R0	{1.85341, 18.0975}	{2.87061, 16.6051}	{{2.76203, 15.3124}, {2.8939, 16.8013}}	{54.8827, 8.33383}	inf	90.	32.64	{11., 62.}	67.5852	0.548908	newly	26.	11.4	{3., 20.}	63.1282	0.49992	Reff	0.615332	0.234882	{0.182316, 0.28939}	61.8284	ar	754.	927.08	{911., 943.}	22.9549
